# Metaverse, virtual reality and augmented reality in total shoulder arthroplasty: a systematic review

**DOI:** 10.1186/s12891-024-07436-8

**Published:** 2024-05-21

**Authors:** Umile Giuseppe Longo, Alberto Lalli, Bruno Gobbato, Ara Nazarian

**Affiliations:** 1 Fondazione Policlinico Universitario Campus Bio-Medico, Via Alvaro del Portillo, 200, Roma, 00128 Italy; 2 Research Unit of Orthopaedic and Trauma Surgery, Department of Medicine and Surgery, Università Campus Bio-Medico di Roma, Via Alvaro del Portillo, 21, Roma, 00128 Italy; 3 Department of Orthopaedic Surgery, Hospital Sao Jose Jaraguá do Sul, Jaraguá, SC 89251-830 Brazil; 4 Musculoskeletal Translational Innovation Initiative, Carl J. Shapiro Department of Orthopaedic Surgery, Beth Israel Deaconess Medical Center, Harvard Medical School, Boston, MA USA

**Keywords:** Metaverse, Augmented reality, Virtual reality, Navigation, Shoulder arthroplasty

## Abstract

**Purpose:**

This systematic review aims to provide an overview of the current knowledge on the role of the metaverse, augmented reality, and virtual reality in reverse shoulder arthroplasty.

**Methods:**

A systematic review was performed using the PRISMA guidelines. A comprehensive review of the applications of the metaverse, augmented reality, and virtual reality in in-vivo intraoperative navigation, in the training of orthopedic residents, and in the latest innovations proposed in ex-vivo studies was conducted.

**Results:**

A total of 22 articles were included in the review. Data on navigated shoulder arthroplasty was extracted from 14 articles: seven hundred ninety-three patients treated with intraoperative navigated rTSA or aTSA were included. Also, three randomized control trials (RCTs) reported outcomes on a total of fifty-three orthopedics surgical residents and doctors receiving VR-based training for rTSA, which were also included in the review. Three studies reporting the latest VR and AR-based rTSA applications and two proof of concept studies were also included in the review.

**Conclusions:**

The metaverse, augmented reality, and virtual reality present immense potential for the future of orthopedic surgery. As these technologies advance, it is crucial to conduct additional research, foster development, and seamlessly integrate them into surgical education to fully harness their capabilities and transform the field. This evolution promises enhanced accuracy, expanded training opportunities, and improved surgical planning capabilities.

## Introduction

The metaverse [1] is a virtual environment that merges physical and virtual realities, empowering users and avatars to interact within a technologically advanced ecosystem [2]. This setting can harness immersive technologies like augmented reality (AR), virtual reality (VR), and artificial intelligence (AI) to provide realistic experiences to individuals across the globe in several different contexts [3].

Computer-driven approaches have been used in many fields of surgery, such as ophthalmology, urology, and general surgery, to assist the surgeon in improving preoperative planning or perfecting surgical execution [4–6]. However, the role of Metaverse, AR, and VR in orthopedics is yet to be adequately elucidated, and their implementation in shoulder surgery is yet to be thoroughly investigated, particularly in the context of shoulder arthroplasty [7]. Several technological innovations are routinely implemented in orthopedic surgery [8], such as robotic surgery, 3D-printed patient-specific instrumentation, and navigation tools with tracking visualized on monitors [9].

The most recent advancement to improve intraoperative execution involves the utilization of computer-assisted navigation instruments. This navigation system offers real-time visual feedback during surgery, enabling precise alignment of the surgeon’s instruments with the preoperative plan. This alignment is achieved by integrating a line-of-sight camera and trackers attached to the surgical instruments and the scapula [10].

While traditional navigation techniques have been extensively utilized in orthopedic procedures, including shoulder arthroplasty, the emerging technologies of AR and VR represent a significant advancement in the field. Notably, there is currently a dearth of studies investigating the application of AR and VR specifically within the context of shoulder arthroplasty, highlighting an area ripe for exploration.

The increasing interest in AR and VR in orthopedics and trauma comes as no surprise, given that orthopedic surgical procedures frequently demand visual data from pre- and intra-operative medical imaging. These procedures involve mechanical actions like screw or implant placements, osteotomies, and deformity corrections, all of which can benefit from visualizing rigid relationships within AR environments. Advancements in haptic feedback, real-time imaging, and AI can further enhance surgical planning, precision, and patient outcomes. Collaborative virtual environments within the metaverse can foster interdisciplinary discussions and enable remote mentoring and guidance for orthopedic surgeons specializing in shoulder procedures [11]. Thus, such technical tasks appear to be predisposed to applications of AR and VR [12].

Also, revolutionary changes in medical education, surgical training, and interventional procedures occur within the metaverse [13]. In this domain, these technologies have the potential to significantly enhance the field of orthopedic surgery by providing a secure and readily accessible supplement to orthopedic surgical training, all without direct involvement of patients [14]. Surgical care and education are increasingly relying on VR, AR, and, ultimately, the newest metaverse applications. Nevertheless, the technologies themselves need further development in this direction, and, at present, it remains challenging to ascertain the extent to which these skills effectively translate into the clinical setting.

The aim of this systematic review is to provide an overview of the current knowledge on the role of the metaverse, AR, and VR in the context of total shoulder arthroplasty.

A comprehensive review of the applications of the metaverse, augmented reality, and virtual reality in *in-vivo* intraoperative navigation, in the training of orthopedic residents, and the latest innovations proposed in *ex-vivo* studies was conducted.

## Materials and methods

### Search strategy

The initial search strategy was organized according to the PICO (Population, Intervention, Comparison, Outcome) structure. Studies that reported outcomes of patients with indications (P) for reverse total shoulder arthroplasty (rTSA) or anatomical total shoulder arthroplasty (aTSA) (I) treated with a computer-assisted intraoperative navigation system were included. Also, studies reporting on orthopedics residents (P) who received VR or AR-based training (I) were included. Cadaver or Computer-based studies (P) reporting outcomes regarding the latest applications of AR or VR on total shoulder arthroplasty (I) were also considered.

Clinical and functional outcomes and questionnaires for each group were reported (C) to evaluate treatment outcomes after each intervention (O).

Two independent reviewers (A.N., A.L) performed article screening using the following research order: title and abstract followed by full article screening. The same reviewers then performed data extraction. In both cases, differences were reconciled by mutual agreement. In case of disagreement, a third reviewer (Longo UG) was consulted for consensus.

### Literature search

A systematic review was performed using the Preferred Reporting Items for Systematic Reviews and Meta-analyses (PRISMA) guidelines. Medline, EMBASE, Scopus, and CINAHL bibliographic databases were searched using the following string: ((metaverse OR augmented reality OR virtual reality)) AND arthroplasty).

The search was performed by two authors (A.L., A.N.) from the inception of the database to August 2023. Additional studies were searched among reference lists of selected papers and systematic reviews.

### Eligibility criteria

The outcomes assessed for patients treated with intraoperative computer-assisted rTSA or aTSA included: the mean number of screws and the mean screw length, the average surgical time, the number and type of augmented baseplates that were exploited, the mean glenoid version and inclination (in its preoperative, planned and postoperative values and the deviation from planned to postoperative glenoid version and inclination. Complications and revisions were also reported.

The following parameters were extracted from the studies that reported on orthopedics residents training with AR or VR and from *in-vitro* studies: the aim of the study, sample size, the instrumentation design, the study results, and conclusions.

To report these variables, peer-reviewed articles of each level of evidence according to the Oxford classification were included. Considering the authors’ proficiency in various languages, articles in English, Italian, French, and Spanish were screened.

Only studies utilizing either computer-assisted intraoperative navigation for rTSA or aTSA were considered. Patients undergoing revision surgery or concomitant procedures were excluded. No exclusion criteria were set regarding the surgical indication or follow-up. Technical notes, letters to editors, and instructional courses were excluded.

Also, only studies reporting outcomes regarding VR- or AR-based training in total shoulder arthroplasty of orthopedic surgical residents were included. Even though they included AR- or VR-based protocols, studies focusing on arthroscopic training were not considered.

### Outcomes of interest

Data was extracted into predefined tables divided according to intervention.

Tables for intraoperative navigated aTSA and rTSA include a demographics table (Table 1), and two outcomes tables (Table 2 and 3.).

**Table 1:** Navigated total shoulder arthroplasty: demographics

AUTHOR AND YEAR	INTERVENTION	GLENOID INCLINATION (Mean °)				GLENOID VERSION (Mean °)				STATISTICALLY SIGNIFICANT FINDINGS	CONCLUSIONS
		Pre-Op	Planned	Post-Op	Deviation	Pre-Op	Planned	Post-Op	Deviation		
**Giorgini et al. 2021**	Navigated rTSA	+ 2.6 ± 6.4 (-7, 18)	-2.7 ± 2.3 (-6, 0)	-2.7 ± 2.3 (-6, 0)	0	-7.6 ± 8.4 (-27, + 2)	-1.6 ± 2.9 (-7, + 3)	-1.6 ± 2.9 (-7, + 3)	0	Surgical time of NAV implants was longer than the surgical time of the last 15 implants performed with no NAV (*p* = .001).	Intraoperative navigation system allows the surgeon to implant the glenoid component with the desired pre-planned positioning with a better accuracy in glenoid and screw positioning. The lack of humeral implant navigation is the main limit of this technique.
**Holzgrefe et al. 2023**	Navigated rTSA	NR				NR				The NAV cohort had better post-op. FE, ER, and CMS.Complications and revisions occurred more commonly in the no NAV cohort .	At early follow-up, NAV RSA compared with No NAV RSA yielded similar rates of improvement in range of motion, functional outcome scores, complications and scapular notching rates.
	Non-Navigated rTSA										
**Hones et al. 2021**	Navigated rTSA	NR				NR				RSAs placed with computer navigation used fewer screws per case (*P* < .001) and had a significantly greater average screw length (*P* < .001).	NAV RSA leads to longer and fewer glenoid baseplate screws being implanted. It appears that computer navigation assists with better screw placement.
	Non-Navigated rTSA										
**Kida et al. 2022**	Navigated rTSA	3.8 ± 3.5	NR	0.3 ± 1.7	NR	4.8 ± 5.5	NR	0.2 ± 1.9	NR	Augmented baseplates were used more frequently in the NAV group than in the no NAV group (*p* = .014). Precision was higher in both version and inclination in NAV group (both *p* < .001).There were more cases of baseplate alignment within 5° of the pre-op. planning in NAV group (*p* = .001 in version and *p* = .001 in inclination). There were fewer cases of baseplate alignment of 8° or more from the pre-op. planning inNAV group (*p* = .010 in version and *p* = .004 in inclination).	The navigation system enables the surgeon to more accurately and precisely reproduce baseplate placement as planned pre-operatively as compared to conventional instrument tion. Furthermore, the navigation system enables real time monitoring of the direction and the use of longer screws.
	Non-Navigated rTSA	4.4 ± 4.8		2.4 ± 6.8		3.8 ± 7.7		-1.0 ± 5.1			
**Kircher et al. 2009**	Navigated rTSA	NR				15.4 ± 5.8 (3, 24)	NR	3.7 ± 6.3 (-8, 15)	NR	The operating time was significantly longer by a mean of 31 min in the NAV group (P 1⁄4 0.001). The correction of retroversion was statistically significant in both groups (*P* < .05). The improvement in accuracy in the NAV group with higher values of correction of retroversion to normal was statistically significant (P 1⁄4 0.021).	The improved accuracy of the glenoid component positioning in the transverse plane using an intraoperative navigation system with greater values of correction to neutral retroversion was validated.
	Non-Navigated rTSA					14.4 ± 6.1 (2, 24)		10.9 ± 6.8 (0.0, 19)			
**Moreschini et al. 2020**	Navigated rTSA	NR			0	NR			0	Cases requiring more than 2 screws to obtain stable primary fixation were significantly lesser in NAV group (p 1⁄4 0.019). Mean screw length was significantly longer in the NAV group (*p* < .001). Significant differences were observed in the use of augmentation between the two groups (p 1⁄4 0.009).	This intra-operative real-time guide allows for going beyond all the problems of surgical exposure of the glenoid, anatomical variability and safety in the positioning of the components.
	Non-Navigated rTSA				0				0		
**Nashikkar et al. 2019**	Navigated RSA or arTSA	5.0 ± 2.6–7.4 (-6, 23)	NR	0.2 ± -1.5-2 (-11, 17)	24.2%	8.2 ± 4.4–11.9 (-6, 43)	NR	1.4 ± 0.4–2.4 (-8, 8)	18.2%	Mean post-op. inclination was significantly superior in the no NAV group. The use of NAV significantly reduced the between-patient variability in post-op. version and led to a significantly greater proportion of components positioned in ‘‘neutral’’ alignment for both inclination (*P* < .01) and version (*P* = .015). Correction from pre-op. glenoid alignment to the post-op. component alignment of the glenoid component was significantly associated (*P* < .01) in both groups for inclination and version. The NAV version correction was significantly more predictable (*P* < .001).	Computer-assisted navigation reduced the average deviation of inclination from neutral and reduced between-patient variability in version postoperatively. Computer assisted navigation has the capacity to replicate the surgical plan in a majority of cases.
	Non-Navigated rTSA or aTSA	4.9 ± 2.2–7.5 (-14, 19)		5.3 ± 3.4–7.2 (-4, 16)		8.4 ± 4.4–12.4 (-19, 36)		-0.2 ± -3.8-3.4 (-28, 22)			
**Rosenthal et al. 2020**	Navigated rTSA or aTSA	NR				NR				There was a statistically significant difference in the operative times using 2D software compared with 3D software for any type of TSA (*P* < .001), aTSAs (*P* < .001), and for rTSAs (*P* < .001). For all types of TSA, there was a statistically significant association between augment use and whether 3D planning was used (*P* < .001).	3D preoperative planning and intraoperative navigation does add time to the surgery, although this additional time was short.
	Non-Navigated rTSA or aTSA										
**Sasaki et al. 2019**	Navigated rTSA	SUP 7.5 ± 8.5 (-9, + 40)	INF 8.0 ± 6.0 (-17, + 3)		4.9 ± 3.8	RET 5.8 ± 10.5 (-15, + 23)		RET 2.0 ± 7.5 (-11, + 9)	5.6 ± 3.6	The post-op. mean inclination of the glenoid component was was significantly different between the groups (P 1⁄4 0.003)	O-arm navigation may improve the accuracy of placement of the inferior tilt of the glenoid component, thus representing a useful tool to improve the inferior tilt of the glenoid procedure in reversed shoulder arthroplasty.
	Non-Navigated rTSA	SUP 11.4 ± 12.8 (-4, + 24)	SUP 5.1 ± 13.6 (-16, + 28)		18.3 ± 11.7	RET 7.0 ± 12.4 (-9, + 24)		ANT 2,9 ± 7.9 (-14, + 10)	7.3 ± 3.6		
**Sprowls et al. 2022**	Navigated rTSA	NR				NR				The NAV and no NAV groups showed statistically significant differences in pre-op. median glenoid retroversion P 1⁄4 0.016) and mean posterior subluxation index. The average individual screw length was significantly higher in the NAV (36.7 mm vs. 30 mm, *P* < .0001) despite the use of significantly fewer screws per case (1, P 1⁄4 0.047). The frequency of augmented baseplate use (*P* < .0001) and the operative time (P 1⁄4 0.001) were significantly higher in the NAV group.	Intraoperative computer navigation allowed for a longer individual screw length, an increased composite screw length, fewer total screws used, and an increased frequency of 2 screws used in total. Preoperative templating software led to a drastic increase in augmented glenoid baseplate use.
	Non-Navigated rTSA										
**Tarallo et al. 2023**	Navigated rTSA	1.7 ± 6.3 (-11, 19)	-2.8 ± 2.3 (-8, 0)	NR		-6.5 ± 6.1 (-20, 6)	-2.1 ± 2.3 (-9, 3)	NR		External rotation showed a twofold increase in mean amplitude from 22° preoperatively to 44.8° at the 2-years follow-up, with 69.6% of patients showing a ROM of 30° or more at follow-up.	Navigation could be a breakthrough to identify the range of lateralization of the glenoid implant.
**Theopold et al. 2019**	Navigated rTSA	NR		-3.2 (-7.4, 3.4)	NR	NR		-1.6 (-14.2, 5.4)	NR	rTSA with no NAV yielded a significant shorter surgery time (*p* < .05).	There is a need for the improvement of 3D image intensifiers algorithms to reduce artifact associated with impaired image quality.
**Wang et al. 2019**	Navigated rTSA	NR			5 ± 3 (0, 11)	NR			3 ± 2 (0, 7)	No learning curve was observed in version accuracy (R2 = 0.083, *P* = .184), with moderate variation observed across the case series. No learning curve was observed in inclination accuracy (R2 = 0.111, *P* = .120). Proficiency in implanting the glenoid component accurately with the planned inclination did not improve with increasing experience.	Intraoperative computer navigation of glenoid component implantation does not increase the total surgical time for rTSA. This system enables placement of the glenoid component with a high level of accuracy and precision. Intraoperative computer navigation is relatively easy to learn, with proficiency that can be acquired after 8 surgical cases.
**Youderian et al. 2023**	Navigated rTSA or aTSA	NR				RET 11 ± 8	NR			Complication rates were not found to be significantly different in any category except for postoperative rotator cuff tear in the aTSA cohort and for dislocations in the rTSA cohort. The internal rotation score (*p* = .003) and external rotation degree (*p* = .001) was significantly higher in the navigated cohort compared to the no NAV aTSA cohort. NAV RSA patients navigated patients had better results in IR, ER, maximum lifting weight, SST and CMS. The use of augmented baseplates was significantly higer in the NAV cohorts.	The use of intraoperative computer-assisted navigation in both ATSA and RTSA is safe, produces at minimum, similar outcomes at two years compared to standard instrumentation, without any increased risk of complications. The potential advantages were more pronounced in rTSA cases.
	Non-Navigated rTSA or aTSA					RET 9 ± 8					

Abbreviations

RCA: Rotator Cuff Arthropathy

RA: Rheumatoid Arthritis

mRCT: Massive Rotator Cuff Tear

PHF: Proximal Humeral Fracture

AVN: Avascular Necrosis

OA: Osteoarthritis

RCC: Retrospective Case-Control

RCS: Retrospective Case Series

rTSA: Reverse Total Shoulder Arthroplasty

aTSA: Anatomical Total Shoulder Arthroplasty

**Table 2:** Navigated total shoulder arthroplasty: outcomes

AUTHOR AND YEAR	INTERVENTION	FOLLOW-UP (Mean, Months)	SCREWS		MEAN SURGICAL TIME (Min (Range))	AUGMENTED BASEPLATES	COMPLICATIONS	REVISIONS
			N° (Mean)	Lenght (Mean, mm)				
**Giorgini et al. 2021**	Navigated RSA	NR	NR	33.5 ± 4.2	92 ± 12 (75–110)	10 Superior 8 Posterior	Coracoid Fracture (1)	None
**Holzgrefe et al. 2023**	Navigated RSA	30.7 ± 7.7	3 (3–4)	NR	NR	108	Glenosphere dissociation (1), Intraop. Humeral Calcar Fracture (1)	1
	Non-Navigated RSA	34.9 ± 9.5	4 (4–4)			57	Infection (1), Loosening (1), Persistent Pain (1), Scapular Fracture, Acromial Fracture (1), Implant Dissociation (1)	4
**Hones et al. 2021**	Navigated RSA	NR	3.4	35.0	NR	0	NR	NR
	Non-Navigated RSA		4.1	32.6		2		
**Kida et al. 2022**	Navigated RSA	NR	4	NR	NR	Posterior 15 Superior 5	NR	
	Non-Navigated RSA		4			6 Posterior 3 Superior		
**Kircher et al. 2009**	Navigated RSA	1.4	NR		169.5 ± 15.2	NR	None	None
	Non-Navigated RSA				138 ± 15.4			
**Moreschini et al. 2020**	Navigated RSA	NR	NR	35.5 ± 4.4	NR	13	NR	
	Non-Navigated RSA			29.9 ± 3.6		4		
**Nashikkar et al. 2019**	Navigated RSA or aTSA	1.4	NR		NR	15	NR	
	Non-Navigated RSA or atSA					6		
**Rosenthal et al. 2020**	Navigated RSA or aTSA	NR	NR		117.9 ± 18.7	54	NR	
	Non-Navigated RSA or atSA				106.44 ± 15.23	15		
**Sasaki et al. 2019**	Navigated RSA	12	NR		192 ± 16.0 (156–214)	NR	None	None
	Non-Navigated RSA				164.6 ± 21.0 (128–191)			
**Sprowls et al. 2022**	Navigated RSA	NR	2.5 ± 0.7	36.7	98.6 ± 19.5	39	NR	
	Non-Navigated RSA		2.8 ± 1	30	85.8 ± 18.7	12		
**Tarallo et al. 2023**	Navigated RSA	24	NR		NR	Posterior 15 Superior 8	Intra-op. Coracoid Fracture (2), GPS Failure (1), Traumatic Dislocations of Implant (2), Infections (2)	4
**Theopold et al. 2019**	Navigated RSA	10–12	NR		126 (104–159)	NR	Intra-op. Coracoid Tracker Malfunctioning (1), Intra-op. Coracoid Tracker Failure (1)	NR
**Wang et al. 2019**	Navigated RSA	NR	3–4	NR	77.3 ± 11.8	NR	None	0
**Youderian et al. 2023**	Navigated RSA	30.9 ± 8.4	NR		NR	NR	Intra-op. Humeral fracture (1), Intra-op. unreported (2), Glenoid loosening (5), RCT (1), Pain (2)	4
	Non-Navigated RSA or atSA	31.3 ± 8.5					Unreported Intra-op. (2), Pain (5), Glenoid Loosening (11), Humeral Loosening (1), RCT (9), Infection (2), Nerve Injury (1)	17

Abbreviations:

rTSA: Reverse Total Shoulder Arthroplasty

aTSA: Anatomical Total Shoulder Arthroplasty

NR: Not Reported

**Table 3:** Navigated total shoulder arthroplasty: outcomes

AUTHOR AND YEAR	STUDY; LOE	AIM	SAMPLE SIZE	INSTRUMENTATION DESIGN	RESULTS	CONCLUSIONS
**Brust et al. 2021**	Proof Of Concept Study; IV	To Present a proof-of-concept system to provide AR guidance during k-wire placement for glenoid component positioning in reversed shoulder arthroplasty, using the Microsoft HoloLens 2 system.	9 Phantom 3D Scapular Models	Microsoft Hololens 2 Device Tornier Aequalis Perform Reversed Implant (Wright Medical Group, USA) Blueprint CT Protocol with Canon Aquilion 64 Scanner mediCAD 3D Shoulder Software (mediCAD Hectec GmbH, GER) mediCAD MR App Stratasys Polyjet 3D Printer (Stratasys, USA) 3D Scanner (Artec Space Spyder, LUX)	The average SD ± error between the planned and achieved entry point was 2.4 ± 0.7 mm. The average SD ± error between the planned k-wire orientation was 3.9 ± 2.4°.	The feasibility of replicating the preoperative CT-based plan was positively demostrated. The use of the high-resolution scanner introduced minimal noise to the measurement of the discrepancy between the planned and achieved position and orientation of the guide wire.
**Darwood et al. 2021**	Basic Science Cadaveric Study; IV	To assess the accuracy and precision of our novel robotic platform for glenoid guidewire placement in the context of total shoulder arthoplasty.	24 Fresh-Frozen Human Cadaver Shoulders	Tableside Robotics Platform: 2-Axis CNC Gimbal + 3-Axis Drill Sterile Disposables: Sterile Guide Blanks Optical 3D Scanner Planning Software (DeSoutter Medical Ltd.)	The first experimental phase achieved end-to-end wire placement accuracy of 1.6° ± 2.4° inclination, 2.2° ± 2.6 version, and 1.2 ± 0.3 mm of wire insertion point accuracy. The second phase achieved end-to-end wire placement accuracy of 1.9° ±1.3° version, 1.2 ± 0.7° inclination, and 1.1 mm ± 0.7 mm of wire insertion accuracy.	This system is able to achieve accuracy levels in keeping with existing technology platforms currently being used in shoulder arthroplasty when assessed in a benchtop cadaver trial.
**Kriechling et al. 2020**	Proof Of Concept Study; IV	To improve and enhance the surgical planning and execution technology using AR and head-mounted display in form of a first feasibility study.	10 3D Phantom Scapular Models	Microsoft Hololens 1 (Microsoft Corp. USA) BF Glenoid Trabecular Metal System (Zimmer Biomet, USA) CT Scan (Siemens Somotom Edge Plus, GER) 3D Printer EOS Formiga P100 (EOS GmbH, GER) CASPA Planning Software (Balgrist CARD, SWI) Unity Software (Unity Technologies, USA) Microsoft Visual Studio (Microsoft Corp. USA)	The mean 3D deviation angle of the ten placed wires measured 2.7° ± 1.3°. The mean deviation to the entry point of the ten placed target wires measured 2.3 mm ± 1.1 mm.	Navigation of the guidewire positioning for the later placement of glenoid components using AR is feasible and accurate.
**Kriechling et al. 2023**	Basic Science Cadaveric Study; IV	To investigate the feasibility of AR navi- gation through HMD to guide the RSA baseplate positioning in a cadaveric study.	12 Fresh-Frozen Human Cadaver Shoulders	Microsoft Hololens 1 (Microsoft Corp. USA) CT Scan (Siemens Somotom Edge Plus, GER) CASPA Planning Software (Balgrist CARD, SWI) Unity Software (Unity Technologies, USA) Microsoft Visual Studio (Microsoft Corp. USA)	The mean deviation from the planned entry point was 3.5 mm ± 1.7 mm. The mean deviation from the planned trajectory was 3.8° ± 1.7°. No adverse event occurred.	The use of AR navigation to position the glenoid baseplate component in RSA is feasible and can achieve good accuracy in a cadaveric setting.
**Rojas et al. 2023**	Basic Science Cadaveric Study; IV	To evaluate the glenoid component placement assisted by AR through an head-mounted display during RSA in cadaveric specimens by analyzing the deviation between the preoperative plan and the postoperative outcomes.	12 Fresh-Frozen Human Cadaver Shoulders	NextAR Navigated Shoulder System (MedActa Internation, SWI) AR Head-Mounted Display MedActa Shoulder Implant System (MedActa International, SWI) CT Scan (Toshiba Aquilion Lightning, JAP) SolidWorks 2016 Software (Dessault Systemes, USA)	The deviations between planned and postoperative values were 1.0° ± 0.7° for inclination, 1.8° ± 1.3° for retroversion, 1.1 ± 0.4 mm for entry point, 0.7 ± 0.6 mm for depth, and 1.7° ± 1.6° for rotation. The deviation between intra- and postoperative measurements were 0.6°± 0.4° for angular measurements and 0.6 ± 0.5 mm for distance measurements. The maximum deviation values between intra- and postoperative mea- surements were 1.5° for inclination and retroversion and 1.6 mm for entry point	The use of a navigated AR system via HMD leads to low deviation between planned and postoperative values in terms of glenoid inclination, retroversion, entry point, depth, and rotation. Additionally, this specific system provides accurate information about the deviation between intraoperative and postoperative values.

Abbreviations:

rTSA: Reverse Total Arthroplasty

aTSA: Anatomical Total Shoulder Arthroplasty

NR: Not Reported

Data from studies focusing on orthopedic surgical residents are reported in Table 4.

**Table 4:** Augmented reality-based training

AUTHOR AND YEAR	INTERVENTION	TYPE OF STUDY	LOE	SAMPLE SIZE	MEAN AGE	INDICATIONS	INSTRUMENTATION DESIGN	TSA SYSTEM
**Giorgini et al. 2021**	Navigated rTSA	RCS	IV	18	75 (62–87)	RCA (7), Concentric Arthritis (4) RA (3), Post Traumatic Arthritis (2) Revision (1), PHF (1), Posterior Luxation (1)	Orthoblue Software Intraoperative GPS	Equinoxe (Exactech, USA)
**Holzgrefe et al. 2023**	Navigated rTSA	RCC	III	113	70.7 ± 7.8	RCA (39), OA (67), mRCT (22)	Intraoperative Exactech GPS Equinoxe Planning App	Equinoxe (Exactech, USA)
	Non-Navigated rTSA			113	7.6 ± 8.1	RCA (45), OA (55), mRCT (33)	None	
**Hones et al. 2021**	Navigated rTSA	RCC	III	100	69.7 (28–87)	OA (51), RCA (43), mRCT (1), Inflammatory Arthropathy (1), Post-Traumatic Arthritis (1), Dislocation Arthropathy (3)	Intraoperative Exactech GPS	Equinoxe (Exactech, USA)
	Non-Navigated rTSA			100	69.3 (49–87)	OA (44), RCA (39), AVN (2), mRCT (1), Inflammatory Arthorpathy (5), Post-Traumatic Arthritis (4), PHF (3), Dislocation Arthropathy (2)	None	
**Kida et al. 2022**	Navigated rTSA	RCC	III	33	75.2 ± 6.4	RCA (NR), mRCT (NR)	Equinoxe Planning App Intraoperative GPS	Equinoxe (Exactech, USA)
	Non-Navigated rTSA			31	75.5 ± 6.1		None	
**Kircher et al. 2009**	Navigated aTSA	RCT	I	10	NR	Osteoarthritis (NR)	Nano Station Optical Tracking System (Praxim, France)	Eclipse (Arthrex, USA)
	Non-Navigated aTSA			10			None	
**Moreschini et al. 2020**	Navigated rTSA	RCC	III	20	75 ± 5.9 (58–84)	Osteoarthritis (NR), RCA (NR)	Exactech Guided Personalized Surgery Software ExactechGPS, BlueOrtho (FRA)	Equinoxe (Exactech, USA)
	Non-Navigated rTSA			20	72 ± 4.9 (64–80)		None	
**Nashikkar et al. 2019**	Navigated rTSA or aTSA	RCC	III	33	71.2 (68–74)	Osteoarthritis (NR), RCA (NR)	Exactech Planning App Exactech GPS	Equinoxe (Exactech, USA)
	Non-Navigated rTSA or aTSA			29	67.4 (64–71)		None	
**Rosenthal et al. 2020**	Navigated rTSA or aTSA	RCC	III	100	69.1 ± 10.1	RCA (NR), Osteoarthritis (NR), RA (NR), mRCT (NR), Osteonecrosis (NR)	Equinoxe Planning App (Exactech, BlueOrtho, FRA) ExactechGPS Total Shoulder Application (Exactech, BlueOrtho, FRA)	Equinoxe (Exactech, USA)
	Non-Navigated rTSA or aTSA			100	68.5 ± 9.1		None	
**Sasaki et al. 2019**	Navigated rTSA	RCC	III	15	77.4 ± 3.2 (71–81)	RCA (8), mRCT (2), RA (3), PHF (2)	Synapse Vincent Image Software (Fujifilm, JAP) StrealthStation S7 Navigation System (Medtronic, USA)	Aequalis Reverse (Tornier, USA)
	Non-Navigated rTSA			10	79.6 ± 7.1 (65–91)	RCA (5), mRCT (2), RA (1), PHF (2), Dislocation Arthropathy (1)	None	
**Sprowls et al. 2022**	Navigated rTSA	RCC	III	51	NR	RCA (106), PHF (3), Hardware Complication (4), Dislocation Arthropathy (1)	Equinoxe Planning App Exactech GPS	Equinoxe (Exactech, USA)
	Non-Navigated rTSA			63			None	
**Tarallo et al. 2023**	Navigated rTSA	RCS	IV	50	73.6 (51–87)	Osteoarthritis (30), mRCT (20)	Orthoblue Software (Exactech, USA) Intraoperative GPS	Equinoxe (Exactech, USA)
**Theopold et al. 2019**	Navigated rTSA	RCS	IV	10	NR	PHF (10)	VectorVision Navigation System (BrainLab AG, GER)	Delta Extend (DePuy Orthopedics, ENG)
**Wang et al. 2019**	Navigated rTSA	RCS	IV	24	73.9 (65–80)	RCA (8) OA (15), Inflammatory Arthritis (1)	BlueOrtho Software (La Tronche, FRA) Exactech GPS	Equinoxe (Exactech, USA)
**Youderian et al. 2023**	Navigated rTSA or aTSA	RCC	III	216	65.5 ± 7.0	RCA (NR), OA (NR), mRCT (NR)	Equinoxe Planning App Exactech GPS	Equinoxe (Exactech, USA)
	Non-Navigated rTSA or aTSA			432	66.0 ± 8.3		None	

Data from in-vitro studies reporting the most recent VR and AR applications in 3D models and cadaver specimens are summarized in Table 5.

**Table 5:** In vitro studies

AUTHOR AND YEAR	STUDY; LOE	AIM	SAMPLE SIZE	INSTRUMENTATION DESIGN	RESULTS	CONCLUSIONS
**Brust et al. 2021**	Proof Of Concept Study; IV	To Present a proof-of-concept system to provide AR guidance during k-wire placement for glenoid component positioning in reversed shoulder arthroplasty, using the Microsoft HoloLens 2 system.	9 Phantom 3D Scapular Models	Microsoft Hololens 2 Device Tornier Aequalis Perform Reversed Implant (Wright Medical Group, USA) Blueprint CT Protocol with Canon Aquilion 64 Scanner mediCAD 3D Shoulder Software (mediCAD Hectec GmbH, GER) mediCAD MR App Stratasys Polyjet 3D Printer (Stratasys, USA) 3D Scanner (Artec Space Spyder, LUX)	The average SD ± error between the planned and achieved entry point was 2.4 ± 0.7 mm. The average SD ± error between the planned k-wire orientation was 3.9 ± 2.4°.	The feasibility of replicating the preoperative CT-based plan was positively demostrated. The use of the high-resolution scanner introduced minimal noise to the measurement of the discrepancy between the planned and achieved position and orientation of the guide wire.
**Darwood et al. 2021**	Basic Science Cadaveric Study; IV	To assess the accuracy and precision of our novel robotic platform for glenoid guidewire placement in the context of total shoulder arthoplasty.	24 Fresh-Frozen Human Cadaver Shoulders	Tableside Robotics Platform: 2-Axis CNC Gimbal + 3-Axis Drill Sterile Disposables: Sterile Guide Blanks Optical 3D Scanner Planning Software (DeSoutter Medical Ltd.)	The first experimental phase achieved end-to-end wire placement accuracy of 1.6° ± 2.4° inclination, 2.2° ± 2.6 version, and 1.2 ± 0.3 mm of wire insertion point accuracy. The second phase achieved end-to-end wire placement accuracy of 1.9° ±1.3° version, 1.2 ± 0.7° inclination, and 1.1 mm ± 0.7 mm of wire insertion accuracy.	This system is able to achieve accuracy levels in keeping with existing technology platforms currently being used in shoulder arthroplasty when assessed in a benchtop cadaver trial.
**Kriechling et al. 2020**	Proof Of Concept Study; IV	To improve and enhance the surgical planning and execution technology using AR and head-mounted display in form of a first feasibility study.	10 3D Phantom Scapular Models	Microsoft Hololens 1 (Microsoft Corp. USA) BF Glenoid Trabecular Metal System (Zimmer Biomet, USA) CT Scan (Siemens Somotom Edge Plus, GER) 3D Printer EOS Formiga P100 (EOS GmbH, GER) CASPA Planning Software (Balgrist CARD, SWI) Unity Software (Unity Technologies, USA) Microsoft Visual Studio (Microsoft Corp. USA)	The mean 3D deviation angle of the ten placed wires measured 2.7° ± 1.3°. The mean deviation to the entry point of the ten placed target wires measured 2.3 mm ± 1.1 mm.	Navigation of the guidewire positioning for the later placement of glenoid components using AR is feasible and accurate.
**Kriechling et al. 2023**	Basic Science Cadaveric Study; IV	To investigate the feasibility of AR navi- gation through HMD to guide the RSA baseplate positioning in a cadaveric study.	12 Fresh-Frozen Human Cadaver Shoulders	Microsoft Hololens 1 (Microsoft Corp. USA) CT Scan (Siemens Somotom Edge Plus, GER) CASPA Planning Software (Balgrist CARD, SWI) Unity Software (Unity Technologies, USA) Microsoft Visual Studio (Microsoft Corp. USA)	The mean deviation from the planned entry point was 3.5 mm ± 1.7 mm. The mean deviation from the planned trajectory was 3.8° ± 1.7°. No adverse event occurred.	The use of AR navigation to position the glenoid baseplate component in RSA is feasible and can achieve good accuracy in a cadaveric setting.
**Rojas et al. 2023**	Basic Science Cadaveric Study; IV	To evaluate the glenoid component placement assisted by AR through an head-mounted display during RSA in cadaveric specimens by analyzing the deviation between the preoperative plan and the postoperative outcomes.	12 Fresh-Frozen Human Cadaver Shoulders	NextAR Navigated Shoulder System (MedActa Internation, SWI) AR Head-Mounted Display MedActa Shoulder Implant System (MedActa International, SWI) CT Scan (Toshiba Aquilion Lightning, JAP) SolidWorks 2016 Software (Dessault Systemes, USA)	The deviations between planned and postoperative values were 1.0° ± 0.7° for inclination, 1.8° ± 1.3° for retroversion, 1.1 ± 0.4 mm for entry point, 0.7 ± 0.6 mm for depth, and 1.7° ± 1.6° for rotation. The deviation between intra- and postoperative measurements were 0.6°± 0.4° for angular measurements and 0.6 ± 0.5 mm for distance measurements. The maximum deviation values between intra- and postoperative mea- surements were 1.5° for inclination and retroversion and 1.6 mm for entry point	The use of a navigated AR system via HMD leads to low deviation between planned and postoperative values in terms of glenoid inclination, retroversion, entry point, depth, and rotation. Additionally, this specific system provides accurate information about the deviation between intraoperative and postoperative values.

General study characteristics extracted were Author, Year of Publication, Type of Study, Level of Evidence (LOE), Intervention, Sample Size, Instrumentation Design, Implant Design, and Last Follow-up.

Outcome measures were extracted from the final follow-up. Mean values and standard deviations were extracted. Depending on the availability of this data from each included study, a selection of these outcomes was included in the tables.

### Methodological quality assessment

The Risk of Bias (RoB 2) tool for Randomized Trials, the Robins-I tool for case-control studies, and the Joanna Briggs Institute Critical Appraisal Tool for Case-Series were used to assess the quality of each study. Two reviewers independently evaluated selected articles (A.L, B.G.) and reviewed by a third in case of disagreement (Longo UG).

## Results

### Study selection

The literature search identified 359 articles from scientific databases and 27 from registers. Duplicate removal resulted in the exclusion of 114 studies, leaving 2 articles for screening.

**Fig. 1:**
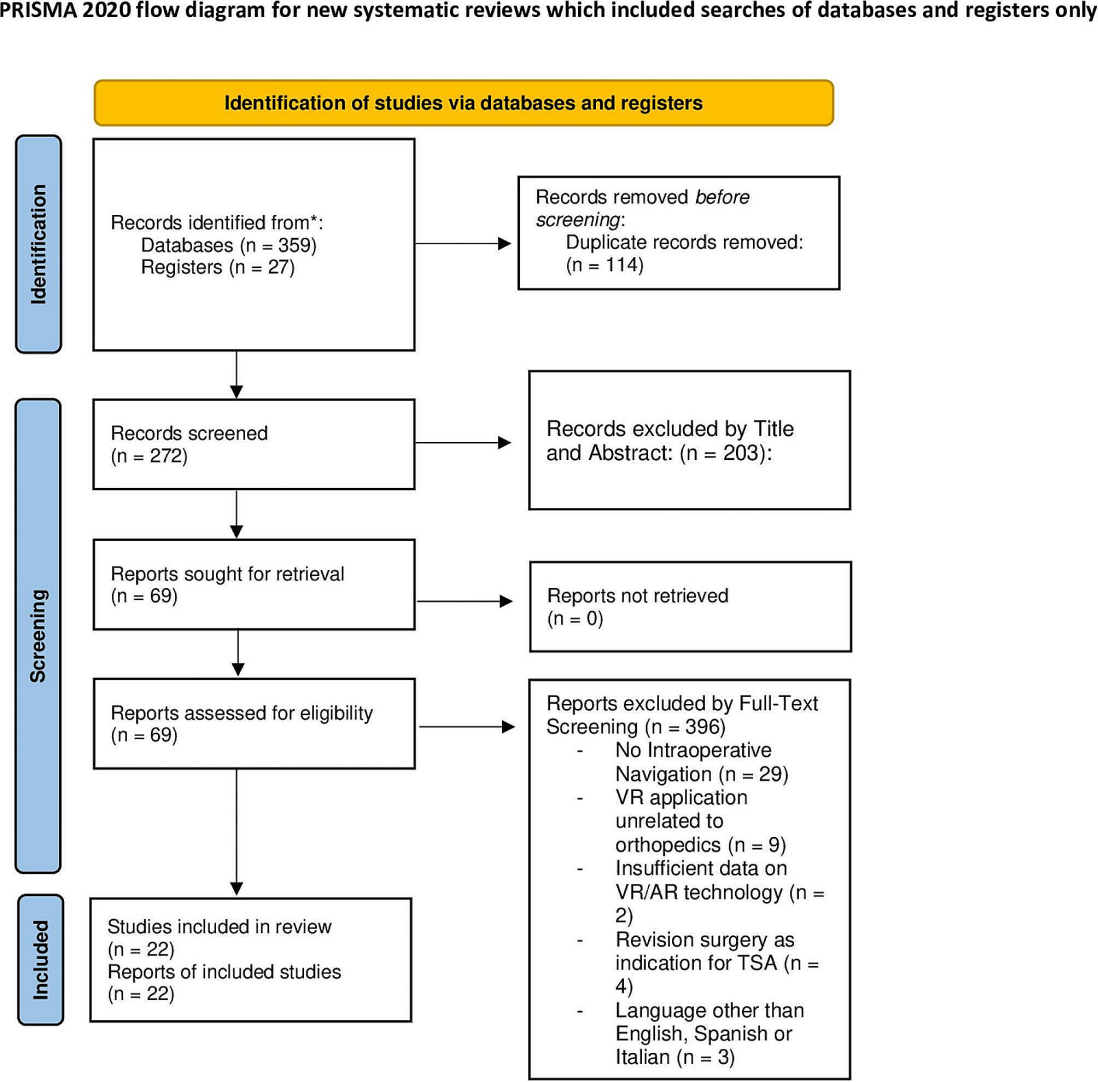
PRISMA flowchart

At the final screening, 22 articles met the selection criteria and were included in the review. The PRISMA flowchart of the literature search is reported in Fig. 1.

**Fig. 2:**
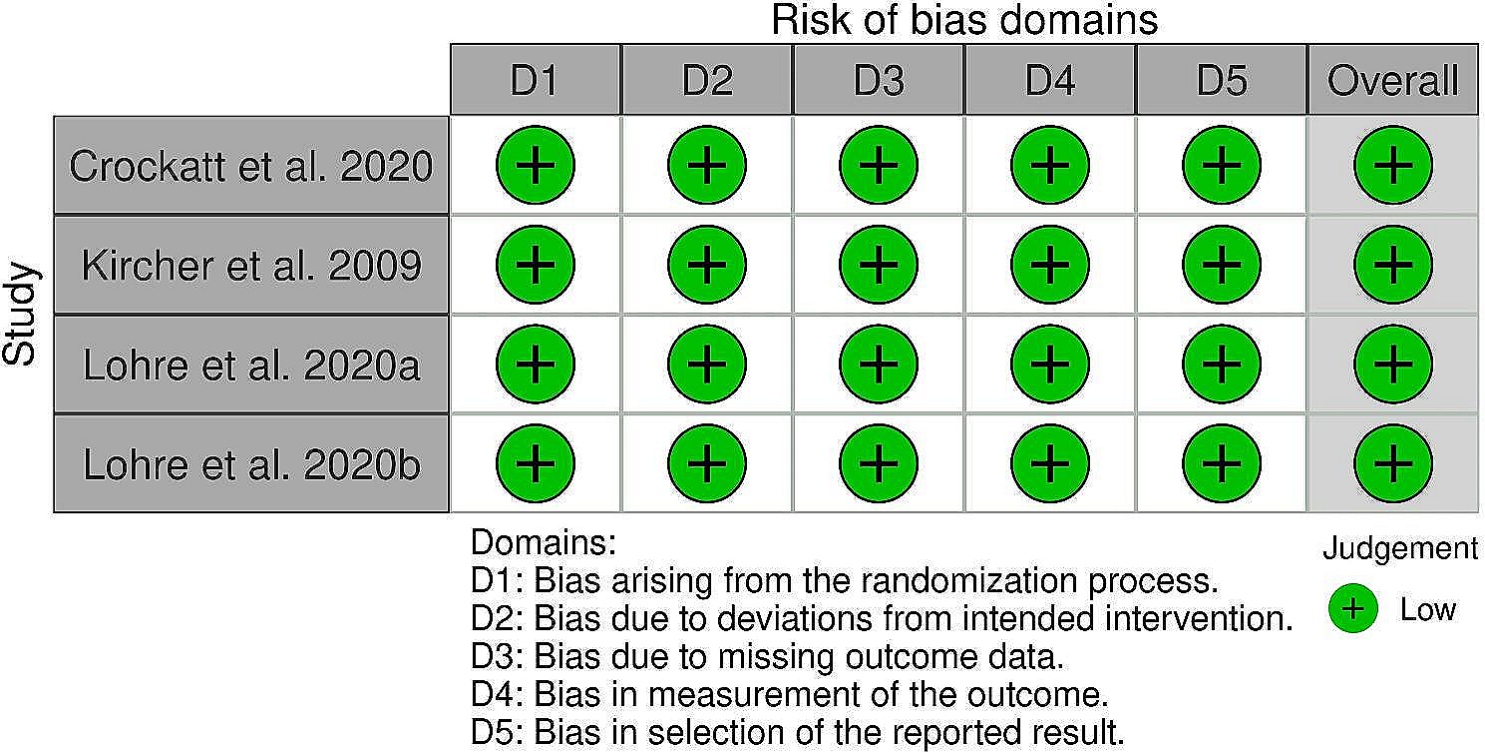
Risk of bias assessment for randomized control trials

**Fig. 3:**
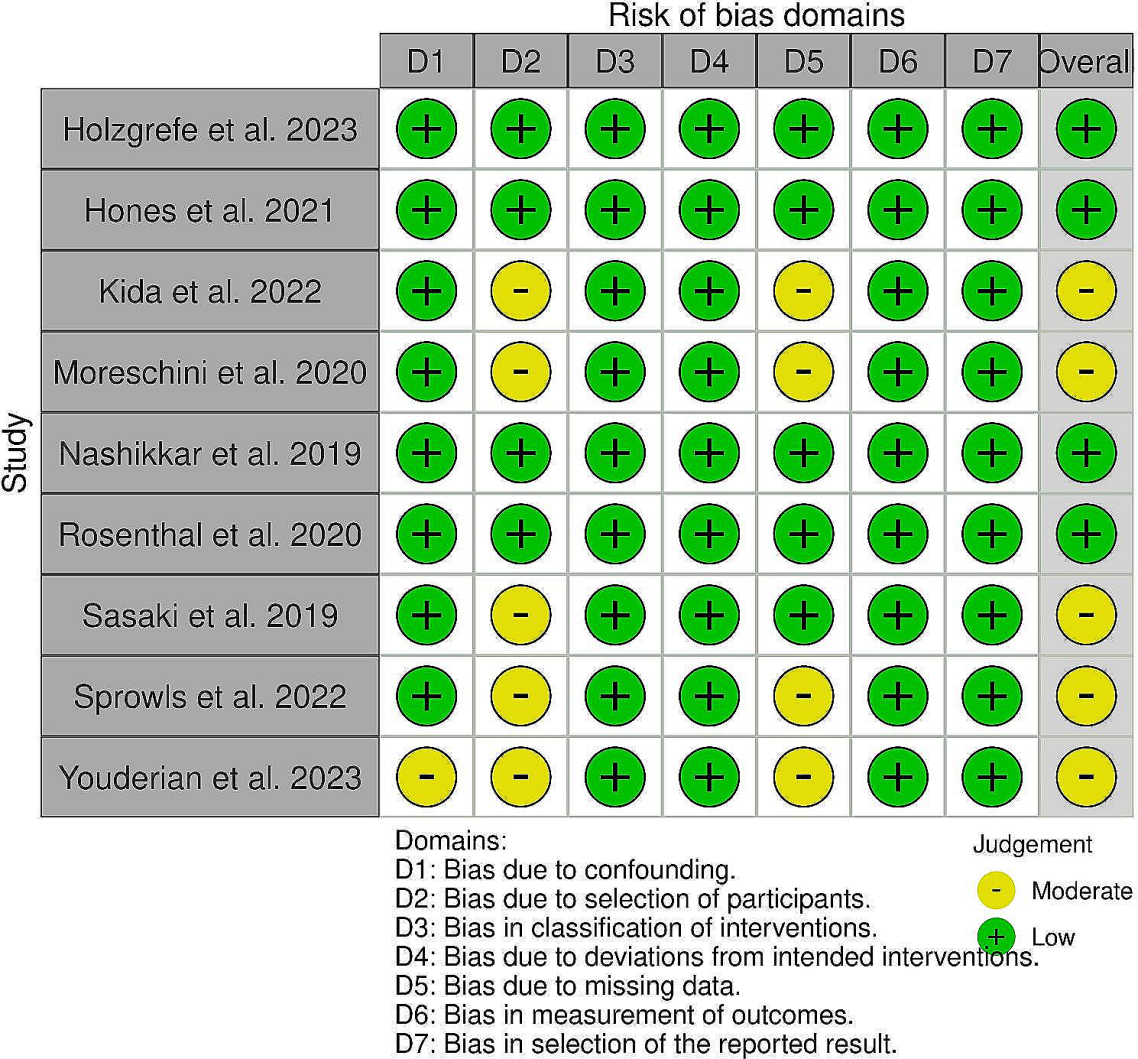
Risk of bias assessment for case-control studies

**Fig. 4:**
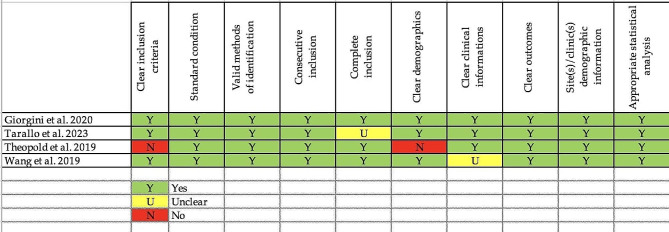
Risk of bias assessment for case series studies

### Study characteristics

The LOE of each of the included studies was: 4 level I Randomized Control Trials [15–18], 9 level III Retrospective Case-Control Studies [19–27], 4 level IV Retrospective Case-Series Studies [28–31], 3 level V Basic Science cadaver studies [32–34] and 2 level V Proof of Concept studies [4, 35].

1701 patients treated with rTSA or aTSA from 14 studies [15, 19–31] were included in the review. 793 patients were treated with navigated rTSA or aTSA, while 908 were treated with standard, non-navigated rTSA or aTSA.

Indications for rTSA and aTSA, whether navigated or non-navigated, included rotator cuff arthropathy, osteoarthritis, massive rotator cuff tears, proximal humeral fractures, osteonecrosis, inflammatory arthropathy, dislocation arthropathy, rheumatoid arthritis, and post-traumatic arthritis. Only one patient in a single study [28] underwent rTSA as a two-stage revision procedure.

The arthroplasty implants included the Equinoxe implant (Exactech, USA), the Eclipse anatomical implant (Arthrex, USA), the Aequalis Reverse implant (Wright Medical Group, USA), and the Delta Extend reverse implant (DePuy Orthopedics, ENG).

Fifty-three orthopedics surgical residents and doctors receiving VR-based training for rTSA were also included in the review. 46 were orthopedics residents from junior to senior years, and 7 were expert orthopedic surgeons. Twenty-seven (23 residents and 4 experts) received VR-based training for rTSA, while 26 (23 residents and 3 experts) were allocated to the cadaver-based training control groups. Data was collected from 3 RCTs [16–18].

In the three studies [32–34] reporting the latest VR and AR-based rTSA applications, 48 fresh-frozen human cadaver shoulders were implanted with the glenoid baseplate via intraoperative navigation integrated with head-mounted displays. Two studies focused on navigated rTSA coupled with a head-mounted display, while a third study exploited a novel robotic platform for glenoid guidewire placement.

Two proof of concept studies [4, 35] reported outcomes following navigated rTSA coupled with the Microsoft Hololens 1 and Hololens 2 devices, used in 19 3D phantom scapulae. The Wright Medical Group Aequalis Reversed Implant (Wright Medical Group, USA) and the BF Glenoid Trabecular Metal System (Zimmer Biomet, USA) were implanted, respectively.

Demographics of patients undergoing navigated and non-navigated rTSA and aTSA are reported in Table 1. Demographics for trainees receiving VR-based training and for *in-vitro* studies are reported in Tables 4 and 5, respectively.

### Quality of evidence

All the included RCTs were judged as “low risk of bias.” Four RCCs were also identified as “low risk of bias,” the remaining three were judged as having a “moderate risk of bias.” CS studies were overall of good quality [36–52]. The Proof of Concept and Basic Science studies were also of adequate quality, even though it was not possible to comment directly on their methodological quality due to the absence of an objective quality assessment measure.

The risk of bias assessments for RCTs, CCs, and CSs are reported in Figs. 2 and 3, and 4, respectively.

### Surgical outcomes

Five studies [19, 20, 23, 24, 30] reported the mean number of screws used in their cohorts, while four studies [20, 21, 23, 28] reported the mean length of the screws used. The mean surgical time was reported by seven studies [15, 23, 25, 26, 28, 30, 31]: the longest time for the navigated and non-navigated cohorts was found by Sasaki et al. [19–26, 29].

Nine articles also reported the number of augmented baseplates used [28].

Complications and revisions were also reported by six articles [19, 27–31]. Common complications included glenoid loosening, persistent pain unexplained by mechanical causes, infection, and intraoperative fractures.

Their preoperative, planned, and postoperative values also reported mean glenoid version and inclination. The mean deviation from the planned and executed glenoid version and inclination were also reported when present in the included articles.

Surgical outcomes for patients undergoing navigated and non-navigated rTSA and aTSA are reported in Table 2and 3.

### VR-based training

One study [16] compared training outcomes for rTSA procedures with iVR platform (PrecisionOS, Canada) as compared with cadaver laboratories among junior orthopedics residents. The VR platform was comprised of a 3D visual tool, auditory cues and handheld controllers for haptic feedback and position tracking. Six residents received the VR-based training and six were enrolled in the control cadaver-based training group. They found no statistically significant differences in written knowledge score, Global Rating Scale (GRS) score, time to completion of assessment, or post-training written knowledge score after implantation of the Reverse Shoulder Augmented Baseplate System (Zimmer Biomet, USA).

A second RCT [18] aimed at determining whether VR training would lead to improved surgical skills in performing rTSA compared to an instructional video in orthopedic surgery residents. Nine residents received the VR-based training and nine were enrolled in the control cadaver-based training group. They found that the VR-trained group had significantly improved Objective structured assessment of technical skill (OSATS) scores as well as higher verbal questioning scores after a single training session.

A third study [17] involved 12 VR-trained residents and surgical experts and 11 residents and experts as controls. They utilized the Glenoid Exposure Model (PrecisionOS, Canada) coupled with a head-mounted display and with haptics tools and found that the immersive VR group completed the cadaveric glenoid exposure task faster as well as demonstrating superior OSATS instrument handling scores compared with the control group.

The outcomes from studies focusing on VR-based training are reported in Table 4.

### In-vitro studies

Two proof of concept studies [4, 35] involving phantom 3D scapular models were included. They aimed to demonstrate a proof-of-concept solution for delivering AR guidance during the placement of k-wires to position the glenoid component in reversed shoulder arthroplasty, employing the Microsoft HoloLens 1 and HoloLens 2 systems. The first one [4] reported that the average standard deviation (SD) ± error between the planned and achieved entry point was 2.4 ± 0.7 mm. The average SD ± error between the planned k-wire orientation was 3.9° ± 2.4°. The other study [35] showed that the mean 3D deviation angle of the ten placed wires measured 2.7° ± 1.3° and that the mean deviation to the entry point of the ten placed target wires measured 2.3 mm ± 1.1 mm.

Three cadaver studies [32–34] were included. They involved twelve, twelve, and twenty-four fresh-frozen shoulders, respectively. They showed that AR-based systems demonstrate accuracy levels consistent with the technology platforms currently employed in shoulder arthroplasty when evaluated in a simulated cadaveric trial.

Outcomes from in-vitro cadaveric and proof of concept studies are reported in Table 5.

## Discussion

The main finding of this systematic review is that intraoperative computer-assisted navigation can attain accuracy levels consistent with the standard technology platforms employed in shoulder arthroplasty. Furthermore, this review shows that VR-based training in rTSA results in comparable if not improved outcomes in surgical skill acquisition in orthopedics residents compared to traditional training protocols. Also, the included cadaveric and proof of concept studies demonstrated that utilizing a navigated AR system through a head-mounted display results in minimal deviation between planned and postoperative values. Furthermore, this system offers precise data regarding the variance between intraoperative and postoperative values.

The integration of emerging technologies such as virtual reality, augmented reality, and the metaverse has ushered in a transformative era in the field of orthopedic surgery [53]. These innovative approaches are shaping the landscape of surgical education and hold substantial clinical relevance within orthopedics, particularly in shoulder surgery [54].

At present, VR is widely recognized for its capacity to develop surgical training simulators and aid in preoperative planning, while AR appears to be a more promising tool for intraoperative purposes [55].

AR use was described as early as 2007 when Ortega et al., who assessed the effects and potential advantages of a heads-up device in spine surgery [56]. Since then, it has been demonstrated that AR could be applied to a wide spectrum of orthopedic procedures, such as tumor resection, fracture fixation, and components alignment in total joint arthroplasty [57].

By projecting 3D models of anatomical structures onto the surgeon’s field of vision, AR can aid in preoperative planning, implant positioning, and intraoperative navigation. Surgeons can visualize patient-specific anatomical landmarks and instrumental paths, ensuring precise alignment during joint replacements and spinal surgeries [58]. AR also enables real-time feedback and guidance, reducing the risk of errors and improving surgical outcomes. Furthermore, AR-based remote collaboration allows experienced surgeons to guide and support less experienced colleagues, enhancing surgical training and fostering interdisciplinary collaboration.

From a technical perspective, the main challenge that must be tackled to make AR a practical instrument for surgery is ensuring the precision of calibration between the virtual content shown by the headset and the actual surroundings. In the context of shoulder replacement, the accurate positioning of the glenoid component has been revealed to be one of the most relevant causes of early revision surgery [15, 21, 23, 30, 59]. To decrease the risk of postoperative aseptic glenoid loosening, understanding the morphology and orientation of the glenoid is a key issue that surgeons must face. Numerous factors have been considered when assessing glenoid stability, including bone density, glenoid morphology, baseplate position, screw length, quantity of peripheral screws, screw angular orientation, and central peg length [60, 61].

The introduction of CT-based preoperative planning software has arguably transformed the mindset of surgeons. Numerous authors have demonstrated that such software enhances a surgeon’s ability to achieve the desired positioning of the glenoid component [28]. However, relying on preoperative 2D analyses has been questioned in terms of accuracy [62–64].

With navigation, the central component of computer-assisted orthopedic surgery systems empowers orthopedic surgeons to precisely monitor and intuitively visualize surgical instruments in real-time within the context of anatomical structures. The human-machine interface, an essential element of image-guided orthopedic navigation systems, is a platform for merging preoperative and intraoperative images from various modalities and three-dimensional models, streamlining operative planning and navigation. The surgeon’s control over the baseplate’s position in terms of version, inclination, rotational alignment, and height is key to enhancing baseplate stability on the native glenoid. Nevertheless, aside from baseplate orientation and bone factors, the number and length of peripheral screws used for primary fixation also play a crucial role in long-term stability [65–68].

A recent systematic review showed that the navigation system increased efficiency in reducing the number of screws necessary for fixation per patient. However, the system’s ultimate clinical and economic impact could not be determined in their study [60].

It has been demonstrated that computer-assisted navigation reduces the deviation of the postoperative component position from the preoperative blueprint in cadaveric studies and in the clinical setting [15, 22, 69–72].

However, while intraoperative navigation has demonstrated enhanced accuracy and precision in glenoid baseplate implantation, there is currently no evidence in the literature to confirm whether these improvements have resulted in better clinical outcomes and reduced complication rates [29, 73]. A recent study showed lower rates of complications and revisions in the navigation group compared to the standard non-navigated procedures. However, it failed to identify increased improvement in range of motion and functional outcome scores compared to the navigated cohort [19].

Another significant factor in glenoid fixation is the number and length of baseplate screws. Before the advent of computer navigation, the capacity to accurately position longer screws was hindered by the difficulty of visualizing the screw’s trajectory due to the absence of clear visual bony reference points. Studies have indicated that increasing the number of screws reduces the likelihood of baseplate displacement, while extending the length of screws may also serve as an effective alternative [20, 65]. A retrospective case-control study showed that computer navigation results in the use of fewer and longer baseplate screws, suggesting that these results may decrease scapular spine stresses and allow for maintained bone stock [20].

While traditional navigation methods have been foundational in guiding surgical procedures, there is a growing recognition of the potential of AR and VR to further enhance surgical precision and improve patient outcomes. Indeed, AR and VR may represent the next evolutionary step beyond traditional navigation techniques. However, it is important to acknowledge that the transition from navigation to AR/VR is not linear, and each technology offers unique advantages and challenges.

AR can combine the advantages of preoperative planning and intraoperative navigation at a low-cost [54]. Following preoperative planning and data transfer to the head-mounted device, the only required intraoperative step is the registration using an optical tracking marker. This surface tracking method eliminates the need for intraoperative imaging, thereby reducing radiation exposure. Kriechling et al. were the first to assess the accuracy and feasibility of guidewire positioning for the placement of glenoid components using AR [35]. The initial outcomes following AR implementation to shoulder replacement surgery were also confirmed by Ponce et al. [74]. Recently, it has been shown that guidewire positioning navigation for placing glenoid components using AR is viable and precise in both cadaver specimens and 3D phantom models [4, 32, 33, 75].

One question is whether AR can replace or improve computer-assisted navigation or robotic-assisted total joint arthroplasty in everyday clinical settings [34]. According to the authors, these novel processes have great potential for transferability to other orthopedic applications in arthroplasty and beyond. As of now, there are no documented clinical applications of AR specifically in shoulder arthroplasty. This underscores the pioneering nature of research in this area and the need for further investigation to explore the potential benefits of AR and VR technologies in improving surgical outcomes in shoulder arthroplasty.

Orthopedic surgical training is also undergoing a paradigm shift [76]. In orthopedic surgical training, the metaverse can provide a collaborative and immersive environment where surgeons, residents, and experts worldwide can interact and learn together [77]. Trainees can participate in virtual surgical conferences, attend live-streamed surgeries, and engage in multidisciplinary discussions. The metaverse offers opportunities for networking, sharing knowledge, and accessing a vast repository of surgical resources. Additionally, the metaverse can facilitate the development of AI-driven surgical assistants, allowing trainees to practice complex procedures with virtual colleagues or receive real-time guidance from virtual mentors [18]. The next logical step would be to systematically employ metaverse, AR, and VR in a training setting. By enabling precise hand-eye coordination, VR fosters the development of surgical skills and has been shown to improve performance in orthopedic procedures such as joint replacements, fracture fixations, and arthroscopic surgeries.

Results have shown that VR-based training significantly reduces surgical errors and enhances surgical proficiency among trainees [78]. Additionally, VR-based simulators offer objective performance metrics, enabling trainees to track their progress and identify areas for improvement.

In a recent investigation, the utilization of AR was assessed for instructing medical students in the placement of acetabular cups for total hip arthroplasty, using a phantom pelvis as the training model [79]. The study revealed that participants exhibited comparable levels of accuracy in their training, whether instructed by an expert surgeon or through AR. Consequently, the authors concluded that the AR approach could be a valuable educational tool, highlighting that certain arthroplasty skills can be acquired without direct supervision [80]. . A recent systematic review [81] has shown that VR-trained residents performed surgery faster and with fewer errors than those trained traditionally. Nonetheless, it has also been shown that VR training significantly improves surgical performance and reduces errors [78].

While it has been demonstrated that AR could offer advantages in training orthopedic residents, it would be intriguing to explore the extent to which AR could truly enhance the learning experience for orthopedic trainees. Furthermore, investigating the learning curve in this context appears to be a promising avenue that warrants further research [82]. However, the training-based application of VR is yet to be fully validated.

The strengths of the present systematic review lie in its novelty: to the authors’ knowledge, this is the first study that provides a comprehensive review of the literature focusing on the applications of AR and VR, as intraoperative computer-assisted navigation, and on the future endeavors that lie in the educational field and technological advancements such as head-mounted displays. Additionally, as per the intraoperative navigation, only primary rTSA or aTSA were included to provide homogeneity of the cohort and improve outcome validation. This review also benefits from using numerous RCTs and including studies with low or moderate risk of bias.

However, there are also limitations associated with the work, including the lack of a meta-analysis, which was not performed given data heterogeneity. Furthermore, indications for total shoulder arthroplasty were not set as exclusion criteria, nor was a minimum follow-up. These limit the validity of the results, particularly on the long-term assessment. Also, the sample size of the VR-based training and cadaveric studies is limited, leaving room for future validation.

## Conclusions

Virtual reality, augmented reality, and the metaverse are transforming the landscape of orthopedic surgery. These technologies provide immersive and interactive platforms that enhance surgical training, improve precision, and advance patient care. By offering realistic simulations, objective feedback, and remote collaboration, virtual reality, augmented reality, and the metaverse hold great promise for the future of orthopedic surgery. As these technologies evolve, further research, development, and integration into surgical education are essential to maximize their potential and revolutionize the field.

## Data Availability

All data generated or analysed during this study are included in this published article [and its supplementary information files].
